# Effect of 4-week physical exercises on tryptophan, kynurenine and kynurenic acid content in human sweat

**DOI:** 10.1038/s41598-021-90616-6

**Published:** 2021-05-27

**Authors:** Tomasz Saran, Monika Turska, Tomasz Kocki, Magdalena Zawadka, Grzegorz Zieliński, Waldemar A. Turski, Piotr Gawda

**Affiliations:** 1 Department of General and Neurorehabilitation, Institute of Rural Health in Lublin, Lublin, Poland; 2 Department of Pharmacology, Faculty of Health Sciences, Medical University of Lublin, Lublin, Poland; 3 Department of Experimental and Clinical Pharmacology, Medical University of Lublin, Lublin, Poland; 4 Department of Sports Medicine, Faculty of Health Sciences, Medical University of Lublin, Lublin, Poland

**Keywords:** Biochemistry, Physiology, Endocrinology, Health care

## Abstract

The aim of the study was the detection of TRP, kynurenine (KYN), and kynurenic acid (KYNA) in human sweat, and determining whether physical activity affects their content in this secrete. Two different methods were used simultaneously—collection of sweat by means of an absorption pad from the inter scapular region, and collection of a drop of sweat from the region of the forehead. Quantitative determinations of TRP, KYN and KYNA were performed using high performance liquid chromatography with ultraviolet and fluorescence detection. Determinations of sodium was carried out by the method of inductively coupled plasma collision/reaction cell ionization mass spectrophotometry. It was found that physical exercises evoked a decrease in the amount of KYN, and an increase in the amount of KYNA in sweat recorded on day 14, but not on day 28 of training. It appears that physical exercises result in a long-term increase in the kynurenine transaminase activity responsible for the formation of KYNA from KYN. Based on this results, it can be suggested that measurement of TRP, KYN and KYNA in sweat may have diagnostic potential and may help to establish an exercise regime appropriate for the age, gender and health status of rehabilitation patients.

## Introduction

Kynurenine (KYN) is a primary metabolite of tryptophan (TRP) formed along the kynurenine pathway. In mammals, the reaction is catalyzed by tryptophan-2,3-dioxygenase (TDO) or indoleamine-2,3-dioxygenases 1 and 2 (IDO). TDO is a constitutive enzyme present primarily in the liver. Its activity is controlled by available TRP, circulating glucocorticoid and heme while feedback inhibition is exerted by NAD(P)H. IDO 1 is an inducible enzyme expressed in almost all extrahepatic cells the activity of which is raised by cytokines during immune activation. Interferon-γ is thought to be a principal effector and nitric oxide is an inhibitor. The role of IDO2 is less known. In comparison to IDO1 it catalyzes TRP degradation under similar condition, but far less efficiently^1,2^. KYN is further metabolized to 3-hydroxykynurenine by kynurenine monoxygenase, anthranilic acid by kynureninase and kynurenic acid (KYNA) by kynurenine aminotransferases^2^. Subsequent bioactive metabolites formed along kynurenine pathway are 3-hydroxyantranilic acid, quinolinic acid and finally nicotinamide adenine dinucleotide^2^.

KYN and its metabolites, collectively called kynurenines, possess diverse biological activities. The role of kynurenines in the central nervous system is a subject of intensive research^3,4^ however, in skeletal muscle is less well understood and remains largely unknown. It was discovered only recently that an intensive transformation of KYN to KYNA takes place in exercising skeletal muscle^5–8^. Currently, it is widely accepted that physical training may exert its beneficial effect on the central nervous system due to the reduction of circulating KYN available for further metabolism in the brain^5,9,10^. KYN is an agonist of aryl hydrocarbon receptor (AhR)^11^. KYNA is an antagonist of glutamate receptors, acting predominantly on N-methyl-D-aspartate (NMDA) receptor. It is also an antagonist of α7-nicotinic acetylcholine receptor and agonist of G protein-coupled receptor 35 (GPR-35) and AhR^12^. The potential role of kynurenines in pathophysiology of the central nervous system has been extensively investigated for more than 3 decades. Initially, it was evidenced that both quinolinic acid and KYNA, glutamate agonist and antagonist, respectively, are involved in the control of neurotransmission, neuronal membrane excitability and neurodegeneration. Afterwards, evidence for association of imbalanced kynurenine pathway with neurological and psychiatric disorders was accumulated. Nowadays, the participation of KYN metabolites in psychoneuroimmunological conditions is advocated^13–15^. Thus, the link between exercise-induced changes in peripheral kynurenine homeostasis and brain functioning appears indisputable^10,16,17^.

KYNA is end metabolite which is excreted mainly with urine^18^. Nevertheless, elevated content of KYNA in plasma may have biological consequences. Since KYNA poorly penetrate the blood–brain barrier^19^ it is unlikely that it affects functioning of the central nervous system. On the contrary, peripheral administration of KYNA exerted antiulcerative activity^20^, reduced inflammation and modulated motility and contractility of the colon^21,22^, increased renal excretion^23^, reduced heart rate^24^ and exerted a cardioprotective effect^25^. Subsequent bioactive metabolites formed along kynurenine pathway are 3-hydroxyantranilic acid, quinolinic acid and finally nicotinamide adenine dinucleotide^2^.

To date, research focused on the effect of physical exercises on kynurenine pathway activity, is based mainly on the measurement of the content of kynurenines and enzymes of the kynurenine pathway in the plasma or tissue collected by means of muscle biopsy^26,27^. Both biopsy and blood sampling are invasive methods and require professional staff and aseptic conditions. As yet, there have been no reports on monitoring the effects of exercise on the level of kynurenines with the use of non-invasive methods.

Since kynurenines were found in secretes such as saliva^28,29^ and human milk^30,31^ it is assumed that they can also be present in sweat. Therefore, the aim of this study was to assess whether TRP, KYN and KYNA are present in a measurable amount in sweat, and to establish the dynamic of changes of their concentrations in human sweat during a 4-week specific physical training in previously untrained individuals.

## Methods

This study was performed in line with the principles of the Helsinki Declaration. Approval was granted by the Ethics Committee at the Institute of Rural Health in Lublin, Poland (Approval No. 3/2015). Study participants were recruited from patients of the Department of General and Neuro-Rehabilitation. The following criteria of inclusion were applied: males and females aged 25–65 years, persons with chronic (> 3 months) mild to moderate low back pain, capable of undertaking moderate endurance training. Eligible patients were informed of the study during their first visit to the out-patient clinic and signed an informed consent before any study-related procedures were performed. A health interview and medical examination were performed before inclusion to the study group. The following exclusion criteria were applied: impaired mobility of joints, which would make it difficult to exercise on devices, permanent or periodic intake of anti-inflammatory and analgesic drugs, duration of ailments less for than four weeks, night or continuous back pain, the presence of sensory symptoms or motor spinal cord injury, systemic inflammatory diseases, obesity, glucose intolerance or diabetes, impaired renal function, heart failure and ischemic heart disease, decompensated hypertension or treatment with diuretics, chronic central nervous system disorders, including mental diseases and history of alcohol or substance abuse, physical or mental disabilities precluding physical testing and/or exercise, and inability to read and understand Polish. A screening instrument was applied to control the state of psychological health—the General Health Questionnaire GHQ-28 D. Goldberg polish adaptation. Only patients with a score lower than 5 were included in the study^32^.

Due to the dietary origin of the pool of TRP, and the crucial involvement of gut microbiota in kynurenine pathway regulations^17^ participants were instructed to continue their eating habits and to inform the investigator about probiotics and medication use throughout the period of the study; however, there were no such reports among participants.

The study group consisted of 35 Caucasian women (n = 25) and men (n = 10), who did not engage in regular physical activity. The mean age was 47.62 years (SD ± 15.91) and the mean Body Mass Index (BMI) was 24.36 kg/m^2^ (SD ± 3.29).

The study participants performed the daily exercise under medical supervision every morning for four consecutive weeks (except Saturdays and Sundays). The two-phase training programme consisted of exercises on a bicycle cycloergometer, followed by exercises on the elliptical cross-trainer. In the first week, the training on each device lasted eight minutes, in the second week it was extended to 10 min, whereas in the third and fourth weeks it took 15 min. There were no breaks longer than 30 s between exercises on both devices. Intensity of the exercise was submaximal. The maximum heart rate was calculated according to the formula: 208–0.7 × age in years. 85% of the maximum heart rate value was assumed as the submaximal value. The exercises were performed at a temperature of 23–24 °C in an air-conditioned gymnasium.

Sweat samples were collected on days one, 14 and 28 of the training. Due to possible errors arising from the specificity of a sweat sampling method, in the present study two different methods of collecting sweat samples were applied: Method A—a commercial absorbent patch (Pharm Chem Inc., USA), and Method B—a cotton bud of commercial ear-tips (Bella Cotton, Poland) were used. Method A: before starting the training session a PharmCheck patch was placed on the skin between the shoulder blades. The patch was removed immediately after finishing the daily training. Method B: a sample (a drop of sweat) was collected from the patient's forehead with a cotton wool swab immediately after finishing the daily training. Next, the pads from patches and the swabs were placed in polyethylene tubes and frozen at −25 °C. After defrosting, 0.5 ml of de-ionized water was added. The solution was separated from pads and swabs by means of centrifugation (4 °C, 15 min, 15,000 rpm) and collected for analyses.

Determinations of plasma sodium were made with a ThermoScientific model XSERIES2 mass spectrometer (USA), with a collision-collision reaction chamber, with ionisation in inductively coupled plasma (ICP-MS). Parameters of the measurement system: nebulizer—glass concentric; spraychamber—glassconical impact bead; Interface option—Xt; sample uptake rate—0.4 mL/min, approx. pumped; quadrupole resolution—standard resolution peak width 0.70 amu at 5% height; plasma gas—Argon (5 grade) 15 L/min; plasma gas CCT—8% Hydrogen in Helium (5 grade) 5 ml/min; sample uptake time—15 s; wash delay—35 s; total time per sample—3 min, 5 s; number of replicates per sample—3. Reagents of purity for trace analysis and deionized water purified with the Millipore Simplicity 185 UV apparatus (USA) were used. The Inorganic Ventures ANALITYK- 122 (USA) pattern was used for calibration of the spectrometer. For the correctness of the calibration curves and for quality control of the performed analyzes, the following certified reference materials were used: EnviroMat ES-L-2 and EnviroMat ES-H-2.

TRP, KYN, and KYNA were measured with high-performance liquid chromatography (HPLC) according to Zhao et al. 2010 with modifications^33^. The HPLC equipment consisted of UltiMate 3000 analytical HPLC system with UltiMate 3000 Autosampler with thermostated column compartment (Thermo Fisher Scientific, Waltham, MA, United States). The samples were kept at a constant temperature (4.0ºC) during the analysis. Chromatography column was set at 30 °C. The HPLC system was equipped with an analytical column (Agilent HC-C18 (2) column; 250 × 4.6 mm i.d.; 5 µm particle size. The mobile phase consisted of 20 mmol/L sodium acetate, 3 mmol/L zinc acetate, and 7% acetonitrile; flow-rate was set at 1 mL/min. All reagents were provided by J. T. Baker and Sigma-Aldrich. The samples were deproteinized with 6% perchloric acid and then centrifuged (30 min, 15,000 rpm). The volume per sample injection was 100 µL. KYN and TRP were measured by UV detection (set for 365 nm and 250 nm, respectively). KYNA was analyzed fluorometrically (excitation 344 nm, emission 398 nm). A Chromeleon software was employed to control the HPLC system and process the chromatographic data.

Data are presented as the content of a determined substance in fmol per µg of Na. Activity of enzymes IDO&TDO and KAT was defined as the ratio of KYN/TRP × 100 and KYNA/KYN, respectively^34^. Data concerning age and BMI of participants are presented as a mean ± standard deviation (SD). The content of sodium, substances in sweat and calculated enzyme activity were expressed as the median with a range of variation (25–75%). Statistical analysis was conducted using Statistica software (ver. 13.1). The statistical comparisons of results were performed using an analysis of variance (ANOVA) for dependent samples with type I error set at 5% and type II error set at 20% (80% power). We determined the minimum number of subjects for adequate study power. The required sample size resulted in N = 26.

The distribution of data was verified using the Shapiro–Wilk test. Intra-group comparisons for normally distributed data were performed using Student's *t* test for dependent samples. For non-normally distributed data, analysis was performed using a non-parametric Wilcoxon rank test. Correlation analyzes, after verifying the linearity, were carried out using the non-parametric Spearman rho correlation coefficient. Statistical significance was accepted at p < 0.05.

## Results

The amounts of Na in the samples collected by the Method A and B on days 14 and 28 did not significantly differ from the value obtained on day one (Table 1).

**Table 1:** Content of natrium (Na), tryptophan (TRP), kynurenine (KYN) and kynurenic acid (KYNA), and activity of indoleamine-2,3-dioxygenase and tryptophan-2,3-dioxygenase (IDO&TDO) and kynurenine aminotransferase (KAT) in human sweat during physical training: upper panel—sampling method A; lower panel—sampling method B.

		1 day	14 day	28 day	Significance	
					14 day vs. 1 day	28 day vs. 1 day
Method A	Na [µg/sample]	183.77 (19.91–529.45)	214.65 (152.52–320.36)	204.89 (100.66–375.46)	Z = 0.57 p > 0.05	Z = 0.37 p > 0.05
	TRP [fmol/µg Na]	586.00 (245.11–1132.98)	763.76 (549.60–1259.84)	892.38 (399.25–1668.04	Z = 0.78 p > 0.05	Z = 1.58 p > 0.05
	KYN [fmol/µg Na]	73.93 (16.47–280.16)	7.44 (3.47–16.09)	15.27 (7.31–52.42)	Z = 3.07 **p < 0.01**	Z = 0.80 p > 0.05
	KYNA [fmol/µg Na]	14.80 (1.30–54.58)	55.64 (27.58 -113.81)	15.26 (8.58–50.01)	Z = 1.29 **p < 0.05**	Z = 0.24 p > 0.05
	KAT	0.12 (0.05–0.15)	6.62 (4.15–8.52)	1.47 (0.37–2.32)	Z = 3.92 **p < 0.001**	Z = 2.93 **p < 0.01**
	IDO/TDO	53.31 (7.75–112.45)	0.92 (0.80–1.20)	2.91 (2.16–4.45)	Z = 4.01 **p < 0.001**	Z = 3.27 **p < 0.05**
Method B	Na [µg/sample]	164.30 (28.75–1136.33)	114.98 (47.60–285.93)	206.53 (57.20–552.80)	Z = 1.25 p > 0.05	Z = 1.53 p > 0.05
	TRP [fmol/µg Na]	448.30 (283.90–1717.62)	925.52 (694.83–3267.29)	473.68 (129.45–8182.81)	Z = 1.59 p > 0.05	Z = 1.01 p > 0.05
	KYN [fmol/µg Na]	34.27 (15.16–677.08)	11.87 (7.44–40.68)	15.47 (7.10–103.24)	Z = 2.44 **p < 0.05**	Z = 0.31 p > 0.05
	KYNA [fmol/µg Na]	8.16 (2.41–53.98)	133.03 (31.32–208.27)	26.60 (8.98–79.06)	Z = 2.56 **p < 0.05**	Z = 0.80 p > 0.05
	KAT	0.12 (0.05–0.29)	6.13 (4.11–8.39)	0.81 (0.22–2.76)	Z = 4.07 **p < 0.001**	Z = 2.58 **p < 0.01**
	IDO/TDO	78.24 (4.12–140.12)	1.10 (0.85–1.28)	2.64 (1.56–3.29)	Z = 3.84 **p < 0.001**	Z = 2.59 **p < 0.01**

Data expressed as the median with a range of variation (25–75%) are presented as the content of determined substance in fmol per µg of Na.

The amounts of TRP in the sweat samples collected by Method A and B on days 14 and 28 did not differ statistically from the values obtained on day one. The content of KYN in sweat samples collected by method A and B on day 14 but not 28 was significantly reduced in comparison to respective control value. The amount of KYNA in the sweat samples collected by Method A and B on day 14 but not 28 was significantly elevated in comparison to respective control value (Table 1).

The activity of KAT in the sweat samples collected by Method A and B on day 14 and 28 was significantly elevated, compared to respective control values obtained on day one. IDO&TDO activity in samples collected by Method A and B on day 14 and 28 was significantly reduced in comparison to control values obtained on day one (Table 1).

## Discussion

The study shows for the first time that metabolites of the kynurenine pathway, KYN, and KYNA, are present in human sweat. TRP was also found in human sweat, although its existence in this secretion was recently announced in a metabolomic study by Cui et al.^35^. It is noteworthy that the current study showed that content of KYN and KYNA varies depending on physical activity, while the amount of TRP in sweat remained unchanged. Interestingly, a large increase in KYNA and significant reduction of KYN content in human sweat was found on day 14 on the physical exercise cycle. On day 28, the differences were insignificant. These findings suggest that physical activity affects kynurenine pathway activity. Therefore, the activity of enzymes responsible for transformation of TRP to KYN and KYN to KYNA were assessed. Enzyme activity was calculated as a ratio of product-to-precursor content. This revealed that IDO&TDO activity was decreased and KAT activity increased throughout the entire exercise cycle. As far as sweat composition reflects the content of substances in blood and tissues, the obtained results point to the conclusion that physical activity leads to the reduction of KYN at the expense of KYNA synthesis.

Saliva seems to be another attractive source of biological material which can be obtained non-invasively. However, it should be considered that presence of KYNA synthetizing enzymes KAT I-III in human saliva and production of KYNA by saliva has been described recently^36^. The stability of kynurenines in such material seems to be a problematic issue. Thus, the usability of saliva for determination of KYNA and other kynurenines as a reflection of their content in the body should be experimentally confirmed.

The results of previous trials in humans studying the effect of physical activity on the kynurenine pathway metabolites agree with the findings of the current study. It should be stressed, however, that in all of the studies the content of kynurenine metabolites formed along the kynurenine pathway was measured in blood. By testing plasma composition in 28 competitors before and after the Boston Marathon, Lewis et al. found an increase of KYNA up to 189% and reduction of TRP up to 23% of initial values^37^. Mudry et al. reported that one-time physical exercises performed on a bicycle cycloergometer increased KYNA and decreased KYN and TRP^6^. Joisten et al. compared the effects of endurance and chronic resistance exercises in healthy adults^34^. They found an enhancement of KYNA and lack of change in KYN content after both exercise interventions; TRP was lowered after resistance but not endurance exercise^35^. The ratio of KYN/TRP and KYNA/KYN was enhanced due to the resistance and endurance activity, respectively. Schlittler et al. showed that endurance training caused an increase in plasma KYNA, while a bout of high-intensity eccentric exercise did not^7^. Taken together, these data confirm that physical activity may affect the metabolism of TRP along the kynurenine pathway; however, the intensity and specific pattern of the alterations differ depending on the nature, extent and duration of the exercise (Table 2). Furthermore, the influence of diet or emotional and mental status has to be taken into account^10,17,38,39^.

**Table 2:** Effects of physical activities on tryptophan, kynurenine and kynurenic acid content in human blood: an overview.

Physical activity	Time	Tryptophan	Kynurenine	Kynurenic acid	Literature
Rugby camp for three days; bicycle ergometry, rugby training, soccer game and 20 km run	post exercise	ND	↑	ND	^40^
	24 h after exercise	ND	=	ND	
Acute graded exercise test on bicycle ergometer	post exercise	↓	↓	↑	^6^
	3 h after exercise	↓	↓	↑	
Cycle ergometer exercise test for 12 weeks	5 min after exercise	↓	=	ND	^41^
Exhaustive aerobic exercise	5 min after exercise	↓	↑	ND	^42^
Acute endurance exercise	post exercise	=	=	↑	^34^
	1 h after exercise	=	=	=	
Resistance exercise	post exercise	↓	=	↑	
	1 h after exercise	=	=	↓	
Half-marathon race	30 min after race	ND	ND	↑	^43^
150 km cycling	1 h after cycling	ND	ND	↑	

↑increase; ↓decrease; = no change; ND—no data.

Nowadays, the pathophysiological relevance of the impact of physical activity exerted on the kynurenine pathway is a matter of debate^16^. On the one hand, the muscle-mediated shift in the balance between KYN and KYNA in blood reduces KYN availability in the brain, and therefore may beneficially affect central nervous system functions^9,10^. On the other hand, the influence of kynurenines on skeletal muscle and peripheral organ should be carefully considered.

Kaiser et al., (2019) presented the hypothesis that activation of AhR by circulating KYN plays a role in muscle loss with aging^44^; indeed, KYN is an agonist of AhR^11^. Experimental data revealed that KYN administration in vivo reduced muscle size and strength in young mice^45^.

The role of KYNA in skeletal muscle morphology and metabolism is unknown. There is only one report showing that KYNA induced protein synthesis and increased the muscle fibres size of C2C12 myoblast cells in vitro. Moreover, KYNA administered in drinking water decreased the loss of muscle mass evoked by limb immobilization in muscle atrophy model in mice^46^. These results were announced in a patent application and not published in a regular peer-reviewed scientific paper.

Unlike in previous studies, we measured the content of TRP, KYN and KYNA in sweat, but not in the blood, because the collection of blood required specialized equipment and assistance of professional personnel. Moreover, it is a painful procedure which is not accepted by many people. Since sweat collection is a non-invasive, painless task, we decided to check its applicability in the determination of kynurenine pathway metabolites during physical activity. Two methods of sweat collection were utilized. A commercial absorbent patch was fixed to the skin (Method A) and commercial ear tips with cotton (Method B) were used. The advantage of Method A is that the patch absorbs sweat throughout the entire test, whereas in the Method B, the drop of sweat was collected at the end of each exercise period, thereby representing temporary sweat content. It is noteworthy that the results obtained by both methods were similar, at least when considering the direction and magnitude of changes in sweat composition. Moreover, the results obtained correspond well with data obtained by other researchers investigating changes of kynurenines in blood^6,34,40–43,47^. Therefore there is reason to conclude that the measurement of TRP, KYN and KYNA in sweat may have the diagnostic potential, and may also help to establish an exercise regime appropriate for the age, gender, and health status of rehabilitation participant. Thanks to the simplicity of the collection of sweat, this procedure may be used to control physical activity on a daily basis according to the strategy of personalized medicine.

The limitation of the present study is that the effect of menstrual cycle was not addressed although number of females exceeded that of males. This issue deserves further detailed analysis, since Hrboticky et al., 1989 reported fluctuation of plasma and urine content of KYN during menstrual period in women orally administered with tryptophan. Interestingly, KYN level was lower in follicular than in luteal phase^48^. This does not correlate to the finding that estrogen(s) increased indoleamine 2,3-dioxygenase and inhibited kynurenine aminotransferases^49,50^.

The another limitation is the absence of a control group and lack of direct correlation between the amount of analyzed compounds in sweat and blood. The control group was not structured. Instead content of tryptophan and kynurenines was individually defined for each participant before the start of exercise cycle and all subsequent measurements were compared to values obtained on day 1. Finally, it should be stressed that our results correlate with data obtained in blood samples by other researchers^6,34,40–43,47^ (Table 2). Evidence is provided that substantial changes in KYN and KYNA content in sweat recorded on day 14 declined or disappeared on day 28 of the exercises. The reason for the dynamics of this phenomenon is not known. It is noteworthy that Millischer et al., 2017, reported unchanged plasma content of KYN and KYNA measured within 1 week after termination of training lasting for 12-weeks. Unfortunately, in this study the content of kynurenines was not determined during the exercises^51^. Certainly, this issue seems to be important because the obtained results may indicate the need for modification of the length, intensity, or set of exercises. It is believed that the use of devices enabling comfortable, non-invasive sweat collection and methods of adequate measurement of kynurenines in secrete can allow easy control and adjustment of rehabilitation processes to individual patient needs.

## Conclusions and practical implications

- TRP, KYN and KYNA is present in detectable amounts in human sweat.
- A two-week cycle of physical exercise decreased the KYN and increased KYNA content in sweat.
- Analysis of the KYN and KYNA sweat content can be considered a useful non-invasive method for monitoring the kynurenine pathway activity during physical training and rehabilitation.
